# Individual and district-level predictors of alcohol use: cross sectional findings from a rural mental health survey in Australia

**DOI:** 10.1186/1471-2458-12-586

**Published:** 2012-08-01

**Authors:** Kerry J Inder, Tonelle E Handley, Michael Fitzgerald, Terry J Lewin, Clare Coleman, David Perkins, Brian J Kelly

**Affiliations:** 1 Centre for Translational Neuroscience and Mental Health, University of Newcastle, Newcastle, NSW, Australia; 2 Hunter Medical Research Institute, Newcastle, NSW, Australia; 3 Centre for Epidemiology and Biostatistics, University of Newcastle, Newcastle, NSW, Australia; 4 Centre for Rural and Remote Mental Health, University of Newcastle, Orange, NSW, Australia; 5 Department of Rural Health, Broken Hill, University of Sydney, Sydney, NSW, Australia

**Keywords:** Alcohol, Mental health, Rural health

## Abstract

**Background:**

Excessive alcohol use is a significant problem in rural and remote Australia. The factors contributing to patterns of alcohol use have not been adequately explained, yet the geographic variation in rates suggests a potential contribution of district-level factors, such as socio-economic disadvantage, rates of population change, environmental adversity, and remoteness from services/population centres. This paper aims to investigate individual-level and district-level predictors of alcohol use in a sample of rural adults.

**Methods:**

Using baseline survey data (N = 1,981) from the population-based Australian Rural Mental Health Study of community dwelling residents randomly selected from the Australia electoral roll, hierarchal logistic regression models were fitted for three outcomes: 1) at-risk alcohol use, indicated by Alcohol Use Disorders Identification Test scores ≥8; 2) high alcohol consumption (> 40 drinks per month); and 3) lifetime consequences of alcohol use. Predictor variables included demographic factors, pre-dispositional factors, recent difficulties and support, mental health, rural exposure and district-level contextual factors.

**Results:**

Gender, age, marital status, and personality made the largest contribution to at-risk alcohol use. Five or more adverse life events in the past 12 months were also independently associated with at-risk alcohol use (Adjusted Odds Ratio [AOR] 3.3, 99%CI 1.2, 8.9). When these individual-level factors were controlled for, at-risk alcohol use was associated with having spent a lower proportion of time living in a rural district (AOR 1.7, 99%CI 1.3, 2.9). Higher alcohol consumption per month was associated with higher district-level socio-economic ranking, indicating less disadvantage (AOR 1.2, 99%CI 1.02, 1.4). Rural exposure and district-level contextual factors were not significantly associated with lifetime consequences of alcohol use.

**Conclusions:**

Although recent attention has been directed towards the potential adverse health effects of district or community level adversity across rural regions, our study found relatively few district-level factors contributing to at-risk alcohol consumption after controlling for individual-level factors. Population-based prevention strategies may be most beneficial in rural areas with a higher socio-economic ranking, while individual attention should be focused towards rural residents with multiple recent adverse life events, and people who have spent less time residing in a rural area.

## Background

Recent reviews have highlighted the significant problem of excessive alcohol use in rural and remote Australia [1]. This pattern is consistent with other high income countries [2]. The factors contributing to high-risk alcohol consumption within rural areas have not been adequately explained [3]. Alcohol use tends to be highest among young adult males and the role of rural culture and heavy alcohol use among men has been identified as important factors to address for the health and welfare of rural men [4]. In addition, recent Australian data suggests worrying levels of alcohol use among older women in rural areas [5]. Furthermore, rural regions in Australia have disproportionately high rates of intentional and non-intentional injury, including motor vehicle accidents and suicide [6], both of which have high Alcohol Attributable Fractions [7]. Given the well established association between these problems and risky drinking, it is particularly important to gain a better understanding of the factors contributing to alcohol use in rural regions.

Some studies have investigated broad individual correlates of high risk alcohol use including age, gender and socio-economic status [8], yet the geographic variation in rates suggest a potential contribution of district-level factors on patterns of alcohol use. District-level factors that may characterise rural and remote communities include socio-economic disadvantage, greater rates of population change, environmental adversity, and remoteness from services/population centres [9].

The Australian Rural Mental Health Study (ARMHS) is a longitudinal population based study established to examine the determinants of mental health in rural and remote communities. Previous multivariate analysis of this cohort has demonstrated that once individual characteristics were accounted for, contextual factors had a minor influence on predicting levels of psychological distress among participants, with evidence for a significant role of social factors (specifically social connectedness and perception of community) [10]. Similar investigation of factors associated with alcohol use, as the primary outcome variable, has the potential to improve knowledge about the factors within rural districts that may contribute to patterns of elevated alcohol use, and to investigate the interaction of person and place based factors in this problem.

Much attention has been given in recent times to the potential adverse health effects of district or community level adversity across rural regions (e.g., drought, perceived financial prosperity and decline of rural community infrastructure) [11,12]. This study aims to investigate these relationships between individual-level factors and contextual district-level factors on alcohol use within rural and remote communities in an Australian setting. We hypothesised that after accounting for the effect of individual characteristics, higher levels of alcohol use would exhibit a significant independent association with rural community factors, based on both individual-level perception of these rural factors and secondary data sources characterising these regions, such as remoteness, regional socio-economic ranking, and environmental characteristics.

## Methods

This paper uses baseline cross sectional ARMHS data to examine the associations of individual, environmental and contextual factors with alcohol use in rural communities. Sixty Local Government Areas were identified from three Australian rural health service regions of the state of New South Wales using the Rural, Remote and Metropolitan Areas classification, representing approximately 70% of the geographic region of non-metropolitan New South Wales. Over-sampling of the remote and very remote regions ensured sufficient sample size from these regions.

The ARMHS baseline sample comprised adults aged 18 years or older living in private dwellings randomly selected from the Australian Electoral Roll. The baseline survey using self report measures, was administered in 2 parts 2 weeks apart (survey A and B) and excluded special dwellings (such as hospitals, nursing homes, prisons, hotels and hostels) and overseas visitors usually resident outside Australia. For further details of ARMHS methodology see Kelly et al., 2010 [10].The project was approved by the Human Research Ethics Committees of the University of Newcastle, University of Sydney, Greater Western Area Health Service, Hunter New England Area Health Service and the North Coast Area Health Service.

### Exclusion criteria

Participants aged 65 years or over were screened for cognitive impairment using the modified Telephone Interview for Cognitive Status [13] and those with a total score < 17 were excluded. Non-English speaking members of a household, those with significant hearing impairment that impeded consent and/or interview, and those with no identifiable telephone contact number (after directory and electronic database search) were also excluded.

### Explanatory variables and instruments

Conceptually-related variables were grouped into 6 domains, reflecting the study’s theoretical interest in the role of pre-dispositional, environmental and contextual factors on alcohol use, with a specific focus on rural-related characteristics. Domains 1–5 were self-reported data from ARMHS baseline postal surveys and for domain 6 data were linked to external sources.

1. Basic demographics

Gender and age in years, categorised into 5 groups (18–34, 35–44, 45–54, 55–64 and 65 and older).

2. Predispositional factors

a. *Trait Neuroticism:* Personality was assessed using the 12 item short form Eysenck Personality Inventory measure of neuroticism (EPQ-N) [14]. A 7-item subset was identified (i.e., being easily hurt, a nervous person, a worrier, being highly strung, suffering from nerves, worrying too long, and often guilty) to conceptually reflect predispositional or trait characteristics, that may be usefully delineated from current distress items, as previously demonstrated by the investigators [15] and others [16].

b. *Level of education:* Categorised as completed high school or not.

c. *Marital status:* Categorised as 1) married or de facto, 2) widowed, 3) never married, and 4) divorced or separated.

3. Recent difficulties and support

a. *Employment status*: Categorised as 1) employed, 2) retired, 3) permanently unable to work, 4) unemployed and 5) student, carer or home duties.

b. *Adverse life events:* A 12 item adverse life events scale was used for events (e.g., relative or friend died; relative ill; argument outside household; demoted or become unemployed; major financial crisis; serious accident; argument within household) within the preceding 12 months [17] and the number of events reported were categorised as 0–2, 3–5 and more than 5.

c. *Perceived prosperity:* was assessed using an item from the Household, Income and Labour Dynamics in Australia study asking about perceived prosperity: ‘Given your current needs and financial responsibilities, would you say that you and your family are: Prosperous, Very comfortable, Reasonably comfortable, Just getting along, Poor or Very Poor?’ [18].

d. *Community and social support*: This score was derived by calculating the mean of the standardised values for the following measures: perceived availability of social support from the Interview Schedule for Social Interaction – Availability of Attachment scale [19], Social Network Index [20], Sense of Community Index [21], and Community Participation Survey [22].

4. Mental health

a. *Current psychological distress*: The Kessler-10 + LM (K-10) [23,24] was used to assess psychological distress and related days out of role during the past 4 weeks.

b. *Recent health service use* for mental health problems was investigated using items from the 2007 Australian National Survey of Mental Health and Well-Being, asking about the number of visits to a range of mental health professionals for mental health problems in the preceding 12 months [25].

c. *Psychiatric disorder*, including alcohol use disorder, was determined using the World Health Organisation Composite International Diagnostic Interview Version 3.0 (WHO-CIDI-3.0) administered by telephone to selected participants based on Kessler-10 score.

5. Rural exposure

a. *Service and support accessibility:* 4 items specifically designed to reflect common concerns in rural communities about infrastructure, including population change (e.g., access to health care or other services, concerns regarding fuel prices, and people moving in or out of the district) were scored on a 5-point Likert scale ranging from ‘not at all’ to ‘a lot’.

b. *Drought related concerns:* A single Likert scale item rating level of worry about drought was collapsed from a 5-point to a 2-point scale, with 1–3 coded as low worry and 4–5 coded as high.

c. *Duration of rural residence:* Participants’ exposure to the specific rural environment was assessed through the number of years residing in the current rural district, expressed as the proportion of life lived in the district.

6. District-level contextual factors

The district-level variables outlined below were obtained from existing databases linked to individual-level data by postcode or Local Government Area extracted from the Australian Bureau of Statistics 2006 census data.

a. *District geographic remoteness category:* The Australian Standard Geographic Classification (ASGC) allocates classes of remoteness to localities, based on the Accessibility/Remoteness Index of Australia Plus (ARIA+): major cities, inner regional, outer regional, remote and very remote. ARIA + index values describe remoteness from goods and services for any part of Australia using road distance as a surrogate for remoteness and the population size of a service centre as a surrogate for the availability of services [26]. This study selected people residing outside major cities and the remaining categories were used for classification of district-level remoteness.

b. *District population change:* This variable represented the percentage change from 2002 to 2006 within the estimated resident population of the Local Government Area, based on birth and death registrations and net migration data obtained from the 2006 Australian Census.

c. *The socio-economic indexes for areas (SEIFA) index of relative socio-economic disadvantage (IRSD)*[27] – is a standardised score based on collation of household level census data (analysed as a continuous variable where higher IRSD scores indicate less disadvantage) chosen for its capacity to provide a postcode level average score representing key dimensions of disadvantage (income, education, employment and household vehicle access).

d. *Drought severity:* The proportion of time out of drought was calculated for the 12 months preceding data collection (using a PostgreSQL database [http://www.postgresql.org] with the PostGIS spatial extension [http://postgis.refractions.net]) by Ivan Hanigan at the National Centre for Epidemiology and Population Health, Australian National University. The Australian Bureau of Meteorology’s gridded monthly rainfall data from 1890–2008, at a resolution of 0.25 degrees latitude-longitude, were used to calculate a drought index based on six-monthly percentiles for each grid cell’s rainfall record [28] averaged within our spatial units, with no weighting by population density [29].

### Alcohol use

The World Health Organization’s Alcohol Use Disorders Identification Test (AUDIT), a 10-item questionnaire, was administered to ARMHS participants by post (as part of survey B) to measure alcohol use. Questions 1–3 measured alcohol consumption, questions 4–6 asked about drinking behaviour and dependence, and questions 7–8 asked about consequences or problems related to alcohol use, all in the last six months.Questions 9–10 ask about lifetime consequences of alcohol use. Responses to each item were scored from 0 to 4, giving a maximum possible score of 40 [30]. Total scores ≥ 8 are indicative of current hazardous alcohol use and scores ≥ 16 of harmful alcohol use in community samples [31,32].

### Data analysis

Data entry, cleaning, aggregation and analysis techniques involved Statistical Package for Social Sciences (SPSS version 17.0; Chicago, IL, USA) and SAS (SAS V9.2; SAS Institute Inc., Cary, NC, USA) statistical software.

The weighting and distribution of AUDIT items are reported initially, together with a validation of AUDIT scores against a WHO-CIDI-3.0 diagnosis of alcohol use disorder, which was administered by telephone to selected ARMHS participants on the basis of screening using current (30 day) psychological distress (Kessler-10).

Alcohol use was dichotomised as low risk (AUDIT score of 0–7) and at-risk (AUDIT score of ≥ 8) [32] and a six-step hierarchical logistic regression analysis across the six domains outlined above was conducted. The order of predictor variables was determined *a priori*, reflecting the presumed order of influence of these variables (e.g., chronological) and their immediacy (e.g., individual versus district-level variables). Aspects of reported personal perception of rural factors were included in the hierarchical models before related district-level characteristics (e.g., the variable of personal concerns about the drought was entered into the model prior to actual duration of drought exposure) to enable investigation of the impact of rurality measures on alcohol use after accounting for individual characteristics. Univariate and multivariate analyses are reported as Odds Ratios (OR) and Adjusted Odds Ratios (AOR) with 99% Confidence Intervals (CI). A higher level of statistical significance (*P* < .01) was used throughout to account for the relatively large sample size and the number of statistical tests undertaken.

As AUDIT scores indicate reported levels of alcohol consumption, drinking behaviour and consequences of drinking, we also explored two components of AUDIT separately to provide more fine grained evaluation of patterns of alcohol use and consequences in this sample. The hierarchical logistic regression was repeated, using the same predictor variables as for the at-risk alcohol use model, for the outcome of high consumption dichotomised as no (≤ 40 drinks per month) versus yes (> 40 drinks per month) calculated using responses to AUDIT questions 1 (How often did you have a drink containing alcohol in the last 6 months?) and 2 (How many drinks containing alcohol did you have on a typical day when you are drinking?). The 40 drinks per month was based on the Australian government National Health and Medical and Research Council (NHMRC) alcohol use guidelines in use at the time the survey was administered recommending no more than 2–4 standard alcohol drinks for men, no more than 2 standard alcohol drinks for women and at least 2 alcohol free days per week (i.e., 10 drinks per week) [33]. A third hierarchical logistic regression investigated lifetime consequences of alcohol use, with participants assigned to the yes category if they answered positively to either question 9 (Have you or someone else been injured as a result of your drinking?) or question 10 (Has a relative or friend or a doctor or other health worker been concerned about your drinking or suggested you cut down?).

## Results

### Response rates and sample biases

Letters of invitation were sent to 13,251 individuals, of whom, 2,639 agreed to participate and completed survey A (participation rate 27%). Participation rates varied across remoteness categories (*χ*_(3)_^2^ = 18.20, *P < .001*), with a marginally higher rate in remote regions (31%). Full details of the sample are described by Kelly et al. [10].

### Sub-sample characteristics

Of the 2,151 participants who completed survey A and B, 170 were excluded from this analysis because of incomplete data, including 26 who did not complete the AUDIT. Those with incomplete data were more likely to be 65 years and older (*P < .001*), female (*P < .01*) and widowed (*P < .001*) and were somewhat less likely to have AUDIT scores of ≥ 8 (*P = .012*). Those with missing data were not different in terms of psychological distress scores (*P = .628*).

Of the 1,981 participants with complete data, 1,694 (85.5%) had an AUDIT score between 0–7, classified as low risk, 233 participants (11.8%) scored 8–15, classified as hazardous level, 33 (1.7%) scored 16–19, classified as harmful, and 21 participants (1.1%) scored 20 or more, classified as high risk. A total of 287 participants (14.5%) reported AUDIT scores ≥ 8, referred to as the at-risk group.

The mean age of those in the at-risk group was younger than those in the low risk group (52 ± 14 *vs.* 55 ± 13 years, *P < .01*) and 67% were men (*P < .001*). More at-risk drinkers were never married (17% *vs.* 5.4% *P < .001*) or divorced or separated (12% *vs.* 9.8%, *P < .001*) and employed (67% *vs.* 53%, *P < .001*) compared to low risk participants. No differences were found according to remoteness category.

### AUDIT item distribution

Table 1 summarises the distribution within the low and at-risk groups for each item of the AUDIT (reported as column percentages). Within the low risk group, AUDIT total scores largely reflected the frequency and quantity of alcohol consumed per occasion (Q1 to Q3), whereas the at-risk group reported additional thoughts, behaviours and consequences (Q4 to Q8), including preoccupation with alcohol (Q4, 43%), guilt (Q8, 35%), and memory lapses (Q6, 32%). Overall, 430 people reported high consumption (> 40 drinks per month), comprising 11% of the low risk group and 86% of the at-risk group. Likewise, 200 people reported at least one lifetime consequence of alcohol use, comprising 3.2% of the low risk group and half (51%) of the at-risk group.

**Table 1:** Distribution of Australian Rural Mental Health Study participants’ responses to items of the Alcohol Use Disorder Identification Test (AUDIT) by total AUDIT score

**AUDIT question**	**Response options**	**Item score**	**AUDIT total score**	
			**0-7 n = 1,694 n (%)**	**> = 8 n = 287 n (%)**
Q1. How often did you have a drink containing alcohol in the last 6 months?	Never	0	389 (23)	
	Monthly or less	1	305 (18)	
	2-4 times a month	2	266 (16)	13 (4.5)
	2-3 times a week	3	323 (19)	59 (21)
	4 or more times a week	4	411 (24)	215 (75)
Q2. How many drinks containing alcohol did you have on a typical day when you are drinking?	1 or 2	0	997 (76)	29 (10)
	3 or 4	1	259 (20)	130 (46)
	5 or 6	2	43 (3.3)	75 (27)
	7 to 9	3	3 (0.2)	41 (15)
	10 or more	4	3 (0.2)	12 (4.3)
Q3. How often during the last 6 months did you have 6 or more drinks on one occasion?	Never	0	978 (75)	22 (7.7)
	Less than monthly	1	232 (18)	54 (19)
	Monthly	2	74 (5.7)	47 (16)
	Weekly	3	21 (1.6)	118 (41)
	Daily or almost daily	4		46 (16)
Q4. How often during the last 6 months have you found it difficult to get the thought of alcohol out of your mind?	Never	0	1,280 (98)	165 (57)
	Less than monthly	1	19 (1.5)	44 (15)
	Monthly	2	3 (0.2)	12 (4.2)
	Weekly	3	3 (0.2)	42 (15)
	Daily or almost daily	4		24 (8.4)
Q5. How often during the last 6 months have you found that you were not able to stop drinking once you had started?	Never	0	1,295 (99)	200 (70)
	Less than monthly	1	9 (0.7)	43 (15)
	Monthly	2	1 (0.1)	14 (4.9)
	Weekly	3		25 (8.7)
	Daily or almost daily	4		5 (1.7)
Q6. How often during the last 6 months have you found that you were unable to remember what happened the night before because you had been drinking?	Never	0	1,286 (99)	195 (68)
	Less than monthly	1	17 (1.3)	72 (25)
	Monthly	2	2 (0.2)	15 (5.2)
	Weekly	3		5 (1.7)
	Daily or almost daily	4		2 (0.7)
Q7. How often during the last 6 months have you needed a first drink in the morning to get yourself going after a heavy drinking session?	Never	0	1,304 (100)	274 (95)
	Less than monthly	1		10 (3.5)
	Monthly	2		1 (0.3)
	Weekly	3	1 (0.1)	1 (0.3)
	Daily or almost daily	4		1 (0.3)
Q8. How often during the last 6 months have you had a feeling of guilt or remorse after drinking?	Never	0	1,267 (97)	186 (65)
	Less than monthly	1	32 (2.5)	57 (20)
	Monthly	2	3 (0.2)	20 (7.0)
	Weekly	3	2 (0.2)	18 (6.3)
	Daily or almost daily	4		6 (2.1)
Q9. Have you or someone else ever been injured as a result of your drinking?	No		1,663 (98)	229 (80)
	Yes, not this year		30 (1.8)	52 (18)
	Yes, this year		1 (0.1)	6 (2.1)
Q10. Has a relative or friend or a doctor or other health worker ever been concerned about your drinking or suggested you cut down?	No		1,665 (98)	168 (59)
	Yes, not this year		27 (1.6)	67 (23)
	Yes, this year		2 (0.1)	52 (18)
*Alcohol consumption greater than 40 drinks per month #*	No		1,511 (89)	40 (14)
	Yes		183 (11)	247 (86)
*Lifetime injury from or someone concerned about your drinking ^*	No		1,639 (97)	142 (49)
	Yes		55 (3.2)	145 (51)

# Based on responses to Q1 and Q2; ^ Based on a Yes response to either Q9 or Q10. Column percentages for Q2 to Q8 are based on respondents who drank during the last 6 months (i.e., excluding Never responses to Q1). Due to rounding, column percentages may not add up to exactly 100%.

As a preliminary check on the validity of the AUDIT scores in the current study, we examined AUDIT distributions for the 517 participants who completed the WHO-CIDI-3.0 alcohol use module, of whom 104 (20%) met criteria for a lifetime alcohol use disorder, including 14 people who met diagnostic criteria for 12-month alcohol use disorder. The mean (SD) AUDIT score of those who did not meet criteria was 3.2 (3.6), compared with 6.1 (5.4) for those who met criteria for lifetime alcohol use disorder, and 14.9 (5.0) for those who met criteria for 12 month alcohol use disorder (*P < .001*). The corresponding median AUDIT scores were 3.0, 5.0 and 14.5, respectively (*P < .001)*.

### Predictors of at-risk alcohol consumption

To facilitate presentation and discussion, the findings from the major six-step hierarchical logistic regression analysis are presented in three tables: steps 1 and 2 (Table 2) – demographic and predispositional factors; steps 3 and 4 (Table 3) – recent difficulties, support, and current mental health; and steps 5 and 6 (Table 4) – rural exposure and contextual factors.

**Table 2:** Hierarchical regression model of at-risk alcohol consumption (AUDIT score ≥ 8) for Australian Rural Mental Health Study participants: steps 1 and 2 - demographic and predispositional factors

**Step - Predictor variable**		**AUDIT total score**		**Univariate OR (99%CI)**	**Multivariate AOR (99%CI)**
		**0-7 n=1,694 n (%)**	**≥ 8 n=287 n (%)**		
1. Demographics					
Age in years	18 - 34	118 (81)	28 (19)		.
	35 - 44	217 (82)	49 (18)	0.95 (0.48, 1.9)	0.90 (0.45, 1.8)
	45 - 54	359 (84)	69 (16)	0.81 (0.43, 1.5)	0.66 (0.34, 1.3)
	55 - 64	476 (84)	93 (16)	0.82 (0.44, 1.5)	0.64 (0.34, 1.2)
	**>=** 65	524 (92)	48 (8.4)	0.39 (0.20, 0.75)**	0.28 (0.14, 0.56)**
Gender	Male	613 (76)	191 (24)		
	Female	1,081 (92)	96 (8.2)	0.29 (0.20, 0.40)**	0.26 (0.18, 0.36)**
2. Predispositional factors					
Trait neuroticism	EPQ-N<3	1,142 (87)	170 (13)		
	EPQ-N>=3	552 (83)	117 (17)	1.4 (1.0, 2.0)*	1.53 (1.1, 2.2)*
Level of (high school) education	Not finished	570 (87)	84 (13)		
	Finished	1,124 (85)	203 (15)	1.2 (0.85, 1.8)	0.97 (0.65, 1.4)
Marital status	Married or defacto	1,302 (87)	197 (13)		
	Divorced or separated	166 (83)	35 (17)	1.4 (0.83, 2.3)	1.3 (0.75, 2.2)
	Widowed	134 (95)	7 (5.0)	0.35 (0.12, 0.96)*	0.69 (0.24, 2.0)
	Never married	92 (66)	48 (34)	3.4 (2.1, 5.7)**	2.9 (1.7, 5.2)**

AOR: Adjusted Odds Ratio; CI: Confidence Interval; EPQ-N: Brief Eysenck Personality Inventory measure of neuroticism; OR: Odds Ratio. Due to rounding, row percentages may not add up to exactly 100%. * *P* < .01, ** *P* < .001.

**Table 3:** Hierarchical regression model of at-risk alcohol consumption (AUDIT score ≥ 8) for Australian Rural Mental Health Study participants: steps 3 and 4 – recent difficulties, support, and current mental health

**Step - Predictor variable**		**AUDIT total score**		**Univariate OR (99%CI)**	**Multivariate AOR (99%CI)**
		**0-7 n=1,694 n (%)**	**≥ 8 n=287 n (%)**		
3. Recent difficulties & support					
Employment status	Employed	898 (82)	192 (18)		
	Retired	585 (90)	63 (9.7)	0.50 (0.34, 0.75)**	0.98 (0.54, 1.8)
	Unable to work	91 (85)	16 (15)	0.82 (0.40, 1.7)	0.64 (0.28, 1.5)
	Unemployed	25 (76)	8 (24)	1.5 (0.51, 4.3)	1.05 (0.30, 3.7)
	Student/carer/home duties	95 (92)	8 (7.8)	0.39 (0.15, 1.04)	0.39 (0.14, 1.1)
Adverse life events	0-2	1,361 (86)	219 (14)		
	3-5	303 (85)	54 (15)	1.1 (0.72, 1.7)	1.1 (0.7, 1.7)
	>5	30 (68)	14 (32)	2.9 (1.2, 6.8)*	3.3 (1.2, 8.9)*
Perceived prosperity: Mean (SD)	3.20 (0.818)	3.14 (0.790)	0.91 (0.75, 1.1)	0.85 (0.67, 1.1)	
Community & social support: Mean (SD)	0.030 (0.676)	-0.110 (0.718)	0.75 (0.60, 0.95)*	1.03 (0.77, 1.4)	
4. Mental health – a) current psychological distress & b) service use					
K-10 category	10-15	1,254 (87)	184 (13)		
	16-24	351 (82)	76 (18)	1.5 (1.0, 2.2)*	1.2 (0.78, 1.9)
	25+	89 (77)	27 (23)	2.1 (1.1, 3.8)*	1.45 (0.68, 3.1)
Contact with a mental health professional #	No	1,366 (87)	212 (13)		
	Yes	328 (81)	75 (19)	1.5 (1.01, 2.2)*	1.2 (0.80, 1.9)

AOR: Adjusted Odds Ratio; CI: Confidence Interval; K-10: Kessler 10 score of psychological distress (30-day); OR: Odds Ratio; # for a mental health problem in past 12 months. Due to rounding, row percentages may not add up to exactly 100%. * *P* < .01, ** *P* < .001.

**Table 4:** Hierarchical regression model of at-risk alcohol consumption (AUDIT score ≥ 8) for Australian Rural Mental Health Study participants: steps 5 and 6 – rural exposure and contextual factors

**Step - Predictor variable**		**AUDIT total score**		**Univariate OR (99%CI)**	**Multivariate AOR (99%CI)**
		**0-7 n=1,694 n (%)**	**≥ 8 n=287 n (%)**		
5. Rural Exposure					
Standardised service and support accessibility: Mean (SD)		-0.029 (1.006)	-0.065 (0.931)	0.96 (0.82, 1.1)	0.98 (0.79, 1.2)
Drought related concerns	Low	1,346 (86)	224 (14)		
	High	348 (85)	63 (15)	1.1 (0.73, 1.6)	1.1 (0.72, 1.8)
Proportion of life lived in a rural district	Whole life	299 (87)	44 (13)		
	≥ half	468 (88)	64 (12)	0.93 (0.54, 1.6)	1.2 (0.66, 2.1)
	< half	927 (84)	179 (16)	1.3 (0.82, 2.1)	1.7 (1.03, 2.9)*
6. Contextual factors					
District remoteness ASGC Category using ARIA+	Inner regional	602 (85)	106 (15)		
	Outer regional	631 (85)	113 (15)	1.02 (0.70, 1.5)	1.2 (0.79, 1.9)
	Remote	329 (86)	52 (14)	0.9 (0.56, 1.4)	1.4 (0.70, 2.8)
	Very remote	132 (89)	16 (11)	0.69 (0.33, 1.4)	0.94 (0.38, 2.3)
District population change 2002-2006 (%): Mean (SD)	0.25 (1.027)	0.39 (0.920)	1.2 (0.97, 1.4)	1.2 (0.89, 1.5)	
Standardised SEIFA IRSD: Mean (SD)	-0.004 (1.019)	0.115 (0.995)	1.1 (0.95, 1.3)	1.1 (0.89, 1.3)	
At least 6 months out of last 12 in drought	No	1418 (85)	256 (15)		
	Yes	276 (90)	31 (10)	0.62 (0.37, 1.05)	0.58 (0.33, 1.03)
District remoteness ASGC Category using ARIA+	Inner regional	602 (85)	106 (15)		

AOR: Adjusted Odds Ratio; ARIA+: Accessibility Remoteness Index of Australia Plus; ASGC: Australian Standard Geographic Classification; CI: Confidence Interval; IRSD: Index of Relative Socioeconomic Disadvantage; OR: Odds Ratio; SEIFA: Socio-Economic Indexes for Area. Due to rounding, row percentages may not add up to exactly 100%. * *P* < .01, ** *P* < .001.

#### Demographic and predispositional factors

As shown in the univariate analyses, participants aged 65 years or older and females had significantly reduced odds of at-risk drinking compared with younger age groups (8.4% *vs.* 19%, OR 0.39, 99%CI 0.20, 0.75) and males (8.2% *vs.* 24%, OR 0.29, 99%CI 0.21, 0.40) respectively. Trait neuroticism was associated with a marginally higher rate of at-risk alcohol use (17% *vs.* 13%, OR 1.4, 99%CI 1.02, 2.0). Widowed participants reported lower alcohol use (5.0% *vs.* 13%, OR 0.35, 99%CI 0.12, 0.96), while participants who were never married reported higher levels of alcohol use (34% *vs.* 13%, OR 3.4, 99%CI 2.1, 5.7). The predictor variables that remained independently associated with at-risk drinking were age, gender, neuroticism, and marital status (i.e., with the never married subgroup reporting higher usage). There were no univariate or multivariate associations between at-risk drinking and level of education.

#### Recent difficulties, support, and current mental health

There were six significant univariate associations between AUDIT at-risk status and the predictors included in steps 3 and 4: fewer retired participants were considered to be at-risk (9.7% *vs.* 18%, OR 0.50, 99%CI 0.34, 0.75); a higher percentage of those with more than 5 adverse events were at-risk (32% *vs.* 14%, OR 2.9, 99%CI 1.2, 6.8); a one standard deviation increment on the community and social support index was associated with a lower odds of being at-risk (OR 0.75, 99%CI 0.60, 0.95); higher current K-10 psychological distress was associated with a higher likelihood of being at-risk (K-10 scores of 16–24: 18% *vs.* 13%, OR 1.5, 99%CI 1.0, 2.2; and K-10 ≥ 25: 23% *vs.* 13%, OR 2.1, 99%CI 1.1, 3.8); as was reporting contact with a mental health professional for a mental health problem in the preceding 12 months (19% *vs.* 13%, OR 1.5, 99%CI 1.01, 2.2). Recent adverse life events was the only predictor variable independently associated with at-risk drinking in steps 3 and 4. The specific life events that were associated with a marginally higher likelihood (*P < .05*) of being in the at-risk group were: becoming unemployed (23% *vs.* 14%); arguments with others in the household (20% *vs.* 14%); and a major financial crisis (20% *vs.* 14%). There were no univariate or multivariate associations between at-risk drinking and perceived prosperity.

#### Rural exposure and district-level contextual factors

There were no significant univariate associations between the rural exposure and district-level contextual factors and at-risk drinking. However, a lower proportion of life spent in the rural district was associated with at-risk drinking (16% *vs.* 13%, OR 1.7, 99%CI 1.03, 2.9) after adjusting for demographic, predispositional, recent difficulties, and mental health factors. Thus, there was no evidence in the current study of associations between at-risk drinking and service or support accessibility, drought related concerns, district remoteness category, district population change, district socioeconomic status, or district drought severity.

### Predictors of high consumption and lifetime consequences

#### High consumption

The hierarchical regression model was repeated for the outcome of *high consumption,* with 430 participants (22%) reporting alcohol consumption of more than 40 drinks per month. In this analysis, high consumption was independently associated with being never married (33% *vs.* 13%, AOR 1.9, 99%CI 1.1, 3.2, *P* < .01) and living in a district with a higher SEIFA ranking (AOR 1.2 per standardised unit, 99%CI 1.02, 1.4, *P* < .01). Female gender was independently associated with lower consumption (13% *vs.* 35%, AOR 0.27, 99%CI 0.20, 0.36, *P* < .001). Lower community and social support was also significantly associated with high consumption at a univariate level, however, this association did not remain after adjusting for demographic and predispositional factors.

#### Lifetime consequences

The hierarchical regression model was also repeated for the outcome of *lifetime consequences,* with 200 participants (10%) reporting at least one lifetime adverse consequence of drinking alcohol. Younger age was significantly associated with lifetime consequences in this model: 21% in those aged 18–34, compared to 11% in those aged 45–54 (AOR 0.39, 99%CI 0.20, 0.78, *P* < .001), 9.8% in those aged 55–64 (AOR 0.35, 99%CI 0.18, 0.68, *P* < .001), and 5.8% in those aged 65 and older (AOR 0.19, 99%CI 0.09, 0.39, *P* < .001). Higher neuroticism (13% *vs* 8.5%, AOR 1.7, 99%CI 1.1, 2.6, *P* < .001) was independently associated with reporting at least one lifetime consequence of alcohol use, while female gender was associated with a lower likelihood of lifetime consequences (7.1% *vs.* 15.0%, AOR 0.39, 99%CI 0.26, 0.58, *P* < .001). Being never married, retired, higher level of education, recent adverse life events, lower community and social support, current psychological distress, and contact with a mental health professional in the past 12 months were significant factors at the univariate level but did not remain significant after adjustment for other factors in the model.

**Figure 1:**
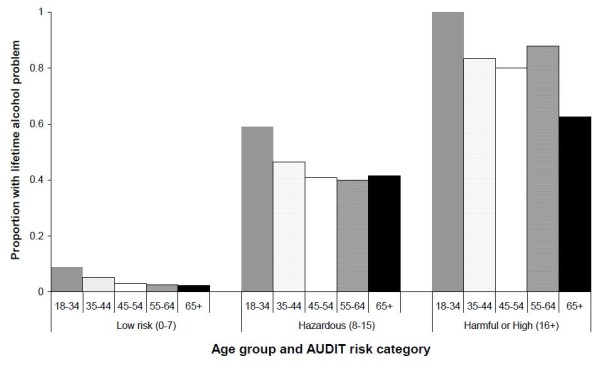
Proportion of Australian Rural Mental Health Study participants with at least one reported lifetime alcohol problem by age group and current Alcohol Use Disorder Identification Test (AUDIT) risk category.

Given the higher than expected rates of lifetime consequences in the youngest age group, this was explored further by examining the proportion of participants in each age group and AUDIT category reporting at least one lifetime consequence; in view of the small sample sizes in some cells, the harmful and high risk categories (total AUDIT score ≥ 16) were combined (see Figure 1). For those in the low risk group, the percentage experiencing at least one lifetime consequence decreased with age, ranging from 8.9% (18–34 years) to 2.4% (65 years and over). For those in the hazardous group, the corresponding percentages were 59% to 42%, while for the combined harmful/high risk group the percentages fell from 100% (18–34 years) to 63% (65 years and older).

## Discussion

Individual-level factors made the largest contribution to problematic alcohol use in this study, with those in the combined ‘at-risk group’ (i.e., hazardous and harmful drinking) being more likely to be male, younger in age, never married, to have higher neuroticism and to have experienced more recent adverse life events. While there was a significant univariate association between levels of psychological distress and alcohol use, this association did not hold in the multivariate analysis once individual predispositional characteristics, recent difficulties and social support were accounted for. The multivariate model for at-risk alcohol use provided limited support for the hypothesis regarding the role of rural specific or regional/district-level factors.

We found patterns of association between adverse life events and at-risk alcohol use, and specifically events indicating interpersonal and financial adversity, although the direction of causation could not be determined from this cross-sectional analysis. This association may be indicative of the adverse consequences of excessive alcohol use, such as becoming unemployed, experiencing a major financial crisis, or close relationship problems. However, this association is also consistent with the identified role of financial stress and economic strain, especially among men, in contributing to adverse health outcomes [34,35] and supports the evidence concerning the link between psychological distress, alcohol use and interpersonal conflict especially among men. Further examination of this relationship is under investigation in the longitudinal follow-up of this sample, which is currently in progress (NHMRC grant #401241).

In addition to at-risk alcohol use, we also examined the outcomes of high consumption and lifetime consequences as measured by the AUDIT. Male gender was associated with all three outcomes. Individual rural exposure factors (e.g., perceived service and support accessibility, worry about drought) were not associated with the outcome in any model in this population, however, with adjustment for other variables a lower proportion of life spent in the rural district was associated with at-risk alcohol consumption. Younger age, neuroticism and increased adverse events were associated with the outcomes of at-risk consumption and lifetime consequences. Never having married was independently associated with at-risk use and high consumption but not lifetime consequences. The district contextual factor of socioeconomic disadvantage was significantly associated with consumption status, such that those with high consumption were likely to be relatively less disadvantaged, reflecting availability of financial resources.

The AIHW 2007 National Drug Strategy Household survey data indicated that at-risk drinking is most prevalent in the 20–29 years age group [36], with reference to the 2001 Australian Alcohol guidelines (NMHRC). Levels of at-risk consumption in this rural study were highest in the 18–34 year age group (approximately one in five) and halved with age (8.4%% in those aged 65 years and older). High consumption varied little by age. However, the younger age group was associated with more lifetime consequences of alcohol (see Figure 1), whereas this may have been expected to increase with age. This result may reflect several possibilities, including the lower response rate from younger males [10]. Alternatively, it may support a genuine cohort effect, reflecting actual societal change, such that drinking related behaviours and incidents involving younger people are viewed more negatively now than previously. On the other hand, it probably represents a simple recall effect, with older people being less likely to recollect past alcohol related incidents, concerns or comments.

National survey data suggest that 20% of Australians (24% of males and 17% of females) consume alcohol at at-risk or high risk levels [36]. Similarly, one in four males in this rural sample reported at-risk alcohol consumption, more than one in three males reported high consumption, and almost one in five males reported at least one adverse lifetime consequence of drinking alcohol. At-risk drinking levels for females in this rural sample were approximately half (8.2%) the rates found in the national data.

With regard to remoteness, at-risk drinking was similar across all remoteness categories in this sample, ranging from 15% in inner and outer regional areas to 14% and 11% in remote and very remote areas respectively. The national data reported that people living in remote or very remote areas were more likely to drink at at-risk or high-risk levels than those living in other areas (32.1% in remote or very remote regions, versus 20.7% in inner regional areas) [36].

ARMHS has measured an extensive range of important variables, including a range of regional/locality data to investigate the factors contributing to variability in alcohol use (covering objective ecological data and subjective, individual perceptions of community/locality). However, this paper was unable to address other known major determinants of alcohol consumption, such as price and physical availability, including distance to alcohol outlets and exposure to advertising and promotion. Estimates of at-risk alcohol use are also potentially subject to selective non-response bias.

The findings generally support the validity of the AUDIT as a measure of clinically significant alcohol problems (in this instance, against concurrent WHO-CIDI-3.0 diagnoses). However, the authors acknowledge the limitations of using the AUDIT for measuring alcohol consumption per se. AUDIT response categories allow only crude estimates of consumption, particularly at higher levels, and are sensitive to measuring current problems as opposed to past problems. For the findings from this study, these limitations potentially impact upon the two alternative regression analyses of high consumption and lifetime consequences. Moreover, although 41% of the AUDIT at-risk group reported drinking 6 or more drinks per occasion on a weekly basis (see Table 1), which is suggestive of a binge drinking pattern, this could not be confirmed without a more detailed alcohol consumption diary.

The cross-sectional nature of this analysis and the low survey response rate limit interpretation of this data, together with our acknowledgement that some factors such as recent adverse life events may be secondary to alcohol use. ARMHS will have longitudinal follow-up data on alcohol consumption at one, three and five year follow-up, allowing the relationship between alcohol use in rural communities to be further explored. The generalisability of this work to the other diverse rural communities, both across Australia and internationally, is also unclear – and awaits replication and refinement – however, younger, single males appear to be a population subgroup deserving specific attention in many communities. Such an emphasis is also compatible with the 12-month prevalence patterns for any alcohol use disorder reported in the 2007 Australian National Survey of Mental Health and Wellbeing (e.g., males, 5.9% *vs.* females, 2.7%; 16–24 year olds, 11.1% *vs.* 65+, 0.7%; single, 9.2% *vs.* married, 1.8%) [37].

## Conclusions

Overall, the findings suggest that younger, single males with a number of recent major adverse events represent the group with the greatest likelihood of hazardous or harmful alcohol use, including experiencing adverse consequences directly related to alcohol consumption. While recent attention has been directed towards the potential adverse health effects of district or community level adversity across rural regions, our study found relatively few district-level factors contributing to at-risk alcohol consumption when individual-level factors were considered. These findings suggest that a comprehensive primary health care approach, with a focus on individual-level factors, particularly male gender, younger age, and adverse life events, to address the elevated levels of at-risk alcohol consumption in rural and remote populations, may be more beneficial than targeting specific district-level factors.

## Abbreviations

AIHW: Australian Institute of Health and Welfare; AOR: Adjusted Odds Ratio; ARIA+: Accessibility Remoteness Index of Australia Plus; ARMHS: Australian Rural Mental Health Study; ASGC: Australian Standard Geographic Classification; AUDIT: Alcohol Use Disorders Identification Test; CI: Confidence Interval; EPQ-N: Brief Eysenck Personality Inventory measure of neuroticism; IRSD: Index of Relative Socioeconomic Disadvantage; K-10: Kessler-10 measure of psychological distress; NHMRC: National Health and Medical Research Council; OR: Odds Ratio; SEIFA: Socio-Economic Indexes for Area; WHO-CIDI-3.0: World Health Organization Composite International Diagnostic Interview version 3.0.

## Competing interests

The authors declare that they have no competing interests.

## Authors' contributions

KJI was involved in the concept and design of the study and interpretation of the data and manuscript preparation. TEH was involved with the statistical analysis and revision of the manuscript. MF performed the statistical analysis and revised the manuscript. TJL participated in the design of the study, the acquisition of data and the analysis and interpretation of data. CC was involved in the design of the study, the acquisition of data and revision of the manuscript. DP participated in the design of the study, the acquisition of data and the revision of the manuscript. BJK conceived the study and participated in its design, contributed to the acquisition and interpretation of data and manuscript revision. All authors have read and approved the final manuscript.

## Pre-publication history

The pre-publication history for this paper can be accessed here:

http://www.biomedcentral.com/1471-2458/12/586/prepub
